# A Rare Case of Page Kidney With Superimposed Infection

**DOI:** 10.7759/cureus.50842

**Published:** 2023-12-20

**Authors:** Emerald Zaw, Jared J Bies, Hein Zay, Eyoab Massebo, Mariam Hassan, Swathi Prakash, Thwe Htay, Mariela Lane

**Affiliations:** 1 Internal Medicine, Paul L. Foster School of Medicine, Texas Tech University Health Sciences Center El Paso, El Paso, USA; 2 Internal Medicine, Texas Tech University Health Sciences Center El Paso, El Paso, USA; 3 Medical Education, Texas Tech University Health Sciences Center El Paso, El Paso, USA

**Keywords:** ace inhibitors and angiotensin receptor blockers, diabetes type 2, resistent hypertension, interventional radiology guided drainage, klebsiella pneumonea, klebsiella kidney abscess, renin-angiotensin-aldosterone system, percutaneous abscess drainage, renal subcapsular hematoma, page kidney

## Abstract

Page kidney (PK) is a rare renal condition characterized by external compression of the kidney, typically by a subcapsular hematoma, leading to resistant secondary hypertension due to hypoperfusion and ischemia. This hypertension is caused by the external compression of the kidney by a chronic subcapsular hematoma that activates the renin-angiotensin-aldosterone system (RAAS) system. Hematoma formation can result from external or internal trauma. The resolution of the hematoma can take months, and, in some cases, may necessitate a nephrectomy. Unresolved subcapsular hematomas can be complicated by infection, leading to sepsis, hospitalization, and the need for surgical drainage. This report presents a unique case of a 67-year-old female with a spontaneous left renal subcapsular hematoma that did not resolve with conservative measures and was complicated by superimposed infection requiring percutaneous drainage.

## Introduction

Page kidney (PK) or Page phenomenon is a rare renal condition characterized by external compression of the kidney, typically by a subcapsular hematoma, leading to resistant secondary hypertension due to hypoperfusion and ischemia [1,2]. The external compression of the kidney by a chronic subcapsular hematoma activates the renin-angiotensin-aldosterone system (RAAS), causing hypertension [1]. The hypertensive response induced by the compression of kidneys was first demonstrated in an animal canine model in 1939 by Irvine Page [2]. A young male football player was the first human case of PK who was found to have a subcapsular hematoma [3]. Understanding the pathophysiology, clinical presentations, and etiologies is crucial to recognizing and treating this condition appropriately.

## Case presentation

A 67-year-old female presented to the emergency department with lethargy, vomiting, lower back pain, flank pain, dysuria, urinary frequency, malodorous urine, and hyperglycemia. Her medical history included insulin-requiring type 2 diabetes mellitus, hypertension, rheumatoid arthritis, and Meniere’s disease. Vital signs were notable for temporal temperature of 39.3 °C, blood pressure of 194/82 mmHg, pulse of 112 beats per minute, respiratory rate of 18 breaths per minute, and oxygen saturation of 93% while she was breathing ambient air. On examination, there was tenderness in the left lower quadrant of the abdomen on palpation without any guarding or rigidity. The curvature of the thoracic and lumbar spine was within normal limits, and the patient exhibited positive left-sided costovertebral angle tenderness. Laboratory test results are shown in Table 1.

**Table 1:** Laboratory data.

Variable	Reference range	Patient results
White blood cells	4.50 x 10^3^ to 11.00 x 10^3^ µL^-1^	12.07 x 10^3^ µL^-1^
Differential count		
Neutrophils	2.00 x 10^3^ to 7.80 x 10^3^ µL^-1^	8.90 x 10^3^ µL^-1^
Lymphocytes	1.00 x 10^3^ to 4.80 x 10^3^ µL^-1^	1.83 x 10^3^ µL^-1^
Monocytes	0.10 x 10^3^ to 1.00 x 10^3^ µL^-1^	1.26 x 10^3^ µL^-1^
Eosinophils	0.00 x 10^3^ to 0.70 x 10^3^ µL^-1^	0.02 x 10^3^ µL^-1^
Basophils	0.00 x 10^3^ to 0.20 x 10^3^ µL^-1^	0.30 x 10^3^ µL^-1^
Hemoglobin	12.0-15.0 G/dL	9.9 G/dL
Hematocrit	36% to 47%	36.8%
Mean corpuscular volume	82-98 fL	95.7 fL
Platelets	150 x 10^3^ to 450 x 10^3^ µL^-1^	222 x 10^3^ µL^-1^
Sodium	135-145 mmol/L	136 mmol/L
Potassium	3.5-5.1 mmol/L	3.7 mmol/L
Chloride	98-107 mmol/L	104 mmol/L
Bicarbonate	22-30 mmol/L	25 mmol/L
Glucose	74-106 mg/dL	93 mg/dL
Blood urea nitrogen	7-17 mg/dL	25 mg/dL
Creatinine	0.52-1.04 mg/dL	1.40 mg/dL
Calcium	8.4-10.2 mg/dL	8.2 mg/dL
Albumin	3.5-5.0 g/dL	2.8 g/dL
Total protein	6.3-8.2 g/dL	5.9 g/dL
Aspartate transaminase	14-36 U/L	18 U/L
Alanine transaminase	0-35 U/L	36 U/L
Alkaline phosphate	38-126 U/L	152 U/L

Urinalysis revealed a small leukocyte esterase, with 21-50 white blood cells per high-power field. The initial chest X-ray indicated a left lower lobe consolidation with pleural effusion. The EKG demonstrated sinus tachycardia. Renal ultrasonography showed no abnormalities. The patient was initially administered intravenous cefepime 1 g q8hr and azithromycin 250 mg for empiric coverage of urinary and respiratory pathogens. A urine culture grew *Candida glabrata*, and the patient’s symptoms of dysuria subsided during the hospital course. The patient’s antibiotic course was narrowed to ceftriaxone 1 g q24hr for coverage of pneumonia.

**Figure 1:**
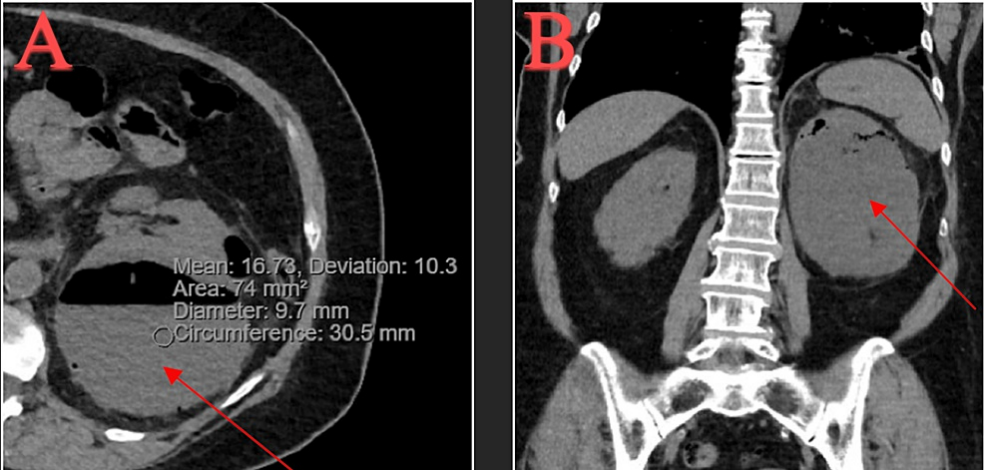
CT abdomen and pelvis w/o contrast of left kidney subcapsular hematoma: (A) transverse view and (B) coronal view. CT, computerized tomography

During hospitalization, a computerized tomography (CT) of the abdomen and pelvis without contrast showed a subcapsular hematoma of the left kidney, measuring 3.8 cm x 5.4 cm x 8.3 cm (Figure 1).

**Figure 2:**
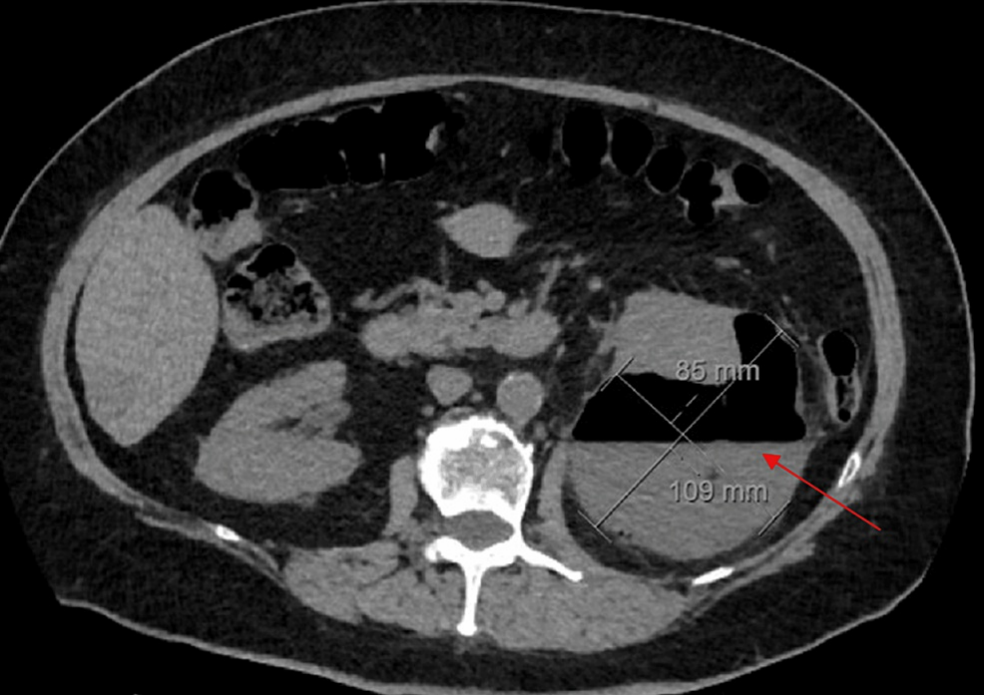
Transverse view of the left kidney subcapsular abscess (measuring 13.3 cm x 10.9 cm x 8.5 cm) on CT abdomen and pelvis without contrast, depicting air-fluid levels within scattered gas foci. CT, computed tomography

Interventional radiology was consulted and recommended outpatient follow-up due to no indication of acute intervention. The patient was discharged with the completion of ceftriaxone and scheduled for outpatient follow-up with a primary care provider. At the initial outpatient follow-up after hospitalization, the patient was found to remain hypertensive despite a multimodal antihypertensive regimen, including amlodipine 10 mg daily and valsartan 40 mg daily. The patient continued to complain of left flank pain. An outpatient CT abdomen and pelvis without contrast was ordered, revealing interval growth of the subcapsular fluid in the posterior aspect of the left kidney, now measuring approximately 13.3 cm x 10.9 cm x 8.5 cm, with the presence of gas foci (Figure 2).

**Figure 3:**
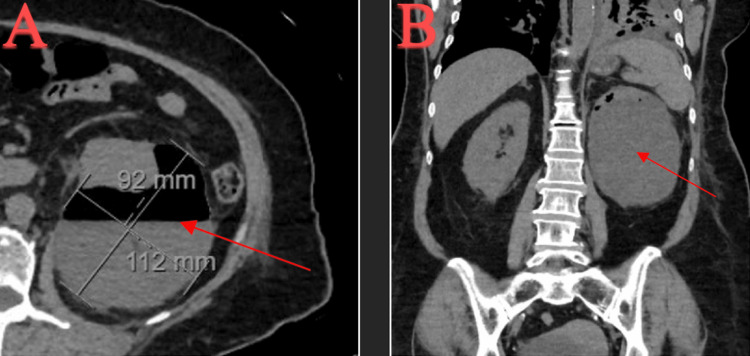
CT abdomen and pelvis w/o contrast: (A) transverse view of a left kidney subcapsular abscess on readmission measuring 11.2 cm x 9.2 cm with air-fluid levels in scattered gas foci and (B) coronal view. CT, computed tomography

The patient had associated fever, chills, and diaphoresis and was advised to go to the hospital. The patient was informed of the risks and benefits of going to the hospital for further assessment and treatment. The patient agreed with hospitalization and presented to the hospital for further treatment. On presentation to the hospital, repeat CT abdomen and pelvis without contrast showed an increased interval size of left renal subcapsular fluid collection measuring 11.2 cm x 9.2 cm, with unchanged loculations and scattered peripheral gas foci (Figure 3).

**Figure 4:**
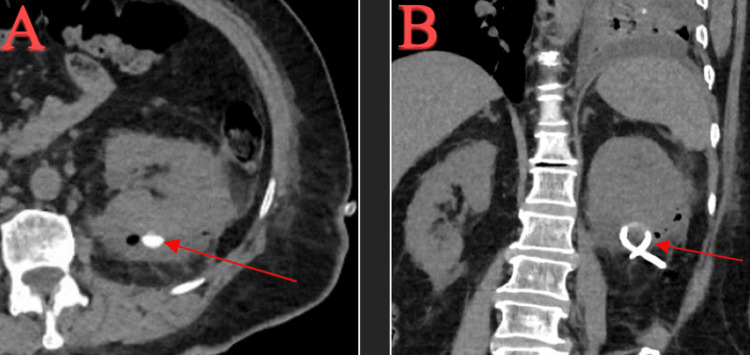
CT abdomen and pelvis w/o contrast of the left kidney subcapsular hematoma with a percutaneous drain in place: (A) transverse view and (B) coronal view. CT, computed tomography

Empiric intravenous piperacillin/tazobactam 4 mg q6hr was started for broad-spectrum coverage. Interventional radiology performed an ultrasound-guided aspiration of the left subcapsular fluid collection. There was no clinical improvement after aspiration and a subsequent CT scan revealed that the size of the left subcapsular renal abscess remained unchanged. A percutaneous drain was placed in the left renal abscess, and approximately 500 mL of pus was drained. A post-procedure CT abdomen and pelvis without contrast, taken two days later, demonstrated near-complete resolution of the abscess (Figure 4).

The patient was initially treated with empiric intravenous piperacillin/tazobactam. Cultures grew *Klebsiella pneumonia*, and antibiotics were narrowed to ceftriaxone based on susceptibility testing. The patient was discharged with oral amoxicillin/clavulanate for the remaining total duration of 20-day antibiotic therapy. After completing the treatment, a three-month follow-up renal ultrasound revealed the resolution of perinephric and subcapsular fluid collections.

## Discussion

PK is a rare etiology of secondary hypertension resulting from chronic subcapsular hematoma-induced external compression of the kidney. This compression triggers the RAAS due to microvascular ischemia and changes in small vessel hemodynamics [4]. This presentation has been recognized as a cause of secondary hypertension for many decades. In 1991, a review of 80 documented cases of PK from 1955 to 1991 was published [5]. In this review of 80 documented cases of PK, the majority of the patients were young male athletes with hypertension and a remote history of blunt trauma to the abdomen or back from participating in contact sports [5]. This review also highlighted the development of PK following a renal biopsy in a patient with underlying IgA nephropathy [5]. Of the 80 documented cases of PK, two-thirds were men, with an average age of 38. The mean elevated blood pressure was 177/95 mm Hg, and there was an elevation in recorded renal vein renin levels lateralized to the affected kidney, supporting the activation of the RAAS as the cause for the increase in arterial blood pressure [4,5]. Since this review, additional cases have been presented in the literature.

The first PK cases consisted of young male athletes with a history of sports-related trauma who developed hypertension [5]. However, in recent years, kidney transplants and biopsies have emerged as new causes of PK in the literature [6,7]. A retrospective study of 518 transplant biopsies, during seven years from January 2000 to September 2007 at a Multi-Organ Transplant Program, London Health Sciences Centre, University Hospital in Ontario, Canada, found an incidence of post-procedure acute PK of about 1% [8]. The increasing incidence of PK from procedural etiologies demonstrates the importance of appropriate treatment to prevent complications. Nontraumatic causes of PK are much rarer, especially in reported literature, and may include tumors, arteriovenous malformations, cyst rupture, glomerulonephritis or vasculitis, subcapsular urinoma, pararenal lymphatic cyst, or idiopathic cases [9,10].

The preferred modality for diagnosing a subcapsular renal hematoma is a CT scan of the abdomen [11]. A CT scan can reveal varying densities of a subcapsular fluid collection, depending on its nature and age [2]. However, it would only be diagnostic of PK if there were concurrent hypertension. A CT with contrast can reveal a delay in contrast excretion, indicating slow renal blood flow that causes RAAS-activated hypertension [12]. Another possible diagnostic indicator of PK in a patient with an appropriate clinical history is reversed diastolic arterial flow in native kidneys [13,14]. This reversed flow is usually seen with renal vein thrombosis of a renal allograft but is less likely in a native kidney because of the rapid formation of capsular venous anastomoses [14]. Therefore, PK should be considered in reversed diastolic arterial flow on a renal Doppler in a patient with hypertension.

Although there are no specific guidelines concerning the treatment of PK, the initial treatment usually involves the management of hypertension through antihypertensive medications, especially those that target the RAAS such as angiotensin-converting enzyme blockers, angiotensin receptor blockers, and aldosterone receptor antagonists [1,6]. Treatment with antihypertensives is initiated to control the patient’s blood pressure and prevent further propagation of the renal subcapsular hematoma. The subcapsular hematoma will typically resolve within three weeks, but if no resolution occurs, the hematoma may require surgical intervention through percutaneous drainage, nephrectomy, or capsulectomy to control hypertension and further complications [15,16].

Common complications of unresolved PK include renal failure, persistent hypertension, hematuria, and loss of renal allograft [1]. Patients with PK are at risk for chronic hypertension, regardless of traumatic or nontraumatic etiology [17]. This may be attributed to perinephric scarring, given that there is often a delay in the injury of the kidney to the onset of the disease [18]. The resulting hypertension may elevate the risk of future cardiovascular events, such as myocardial infarction and stroke, making it one of the reasons for prompt management of the underlying cause and pathologically high blood pressure. Surgical interventions such as percutaneous drainage of the subcapsular hematoma have been shown to reverse worsening kidney function and elevated blood pressure [19]. Early detection could potentially avoid the need for surgical intervention to treat PK [20].

## Conclusions

This study presents a unique case of a patient who presented with acute PK secondary to an acute spontaneous idiopathic left renal subcapsular hematoma that became infected. Although most cases have been associated with traumatic etiologies, current literature supports rare cases of spontaneous idiopathic renal subcapsular hematoma formation leading to PK. Literature shows a paucity of such cases, and this is a rare presentation of complications that can arise once a diagnosis of PK is established. Physicians must be aware of other reversible causes of hypertension such as uncontrolled pain, anxiety, poorly managed diabetes mellitus, and alcohol or narcotic withdrawals before pursuing a workup for PK. Once all these have been appropriately ruled out and the patient continues to have resistant hypertension, a physician who has high clinical suspicion for PK can pursue CT imaging of the abdomen to rule out the suspected diagnosis and guide treatment. This case presentation serves as another supplemental piece of literature for practicing physicians to understand the need for appropriate and timely recognition and management of a subcapsular hematoma causing PK to prevent infections and further sequelae.
